# Adverse Effects of COX-2 Inhibitors

**DOI:** 10.1100/tsw.2005.82

**Published:** 2005-08-18

**Authors:** Jagdish N. Sharma, Najeeba M. Jawad

**Affiliations:** Department of Applied Therapeutics, Faculty of Pharmacy, Health Sciences Centre, Kuwait University, P.O. Box 24923, Safat 13110, Kuwait

**Keywords:** prostaglandins, nonsteroidal anti-inflammatory drugs, cyclooxygenase inhibitors, cardiovascular effects, adverse effects, COX-2 inhibitors

## Abstract

Cyclooxygenase-2 selective inhibitors (COXIBs) were developed with the prime object of minimizing gastrointestinal adverse effects, which are seen with the use of traditional nonsteroidal anti-inflammatory drugs (NSAIDs). Their long-term use is limited by the development of hypertension, edema, and congestive heart failure in a significant proportion of patients. NSAIDs block the activity of both COX isozymes, COX-1 and COX-2, which mediate the enzymatic conversion of arachidonate to prostaglandin H_2_ (PGH_2_) and other prostaglandin (PG) metabolites. It is well established that the cardiovascular profile of COX-2 inhibitors can be accounted for by inhibition of COX-dependent PG synthesis. Following the COX-mediated synthesis of PGH_2_ from arachidonate, PGH_2_ is metabolized to one of at least five bioactive PGs, including PGE_2_, PGI_2_, PGF_2_, PGD_2_, or thromboxane A_2_ (TXA_2_). These prostanoids have pleiotropic cardiovascular effects, altering platelet function and renal function, and they are acting either as vasodilators or vasoconstrictors. Although COX-1 and COX-2 exhibit similar biochemical activity in converting arachidonate to PGH_2_*in vitro*, the ultimate prostanoids they produce *in vivo* may be different due to differential regulation of COX-1 and COX-2, tissue distribution, and availability of the prostanoid synthases. PGs have been established as being critically involved in mitigating hypertension, helping to maintain medullary blood flow (MBF), promoting urinary salt excretion, and preserving the normal homeostasis of thrombosis, and the researchers found that the use of COX-2 inhibitors caused many serious complications in altering the normal body homeostasis. The purpose of the present research is to explain briefly the side effects of COX-2 inhibitors on the renal and cardiovascular system.

### INTRODUCTION

Prostaglandins (PGs) are any group of potent, hormone-like substances that are produced in various mammalian tissues, derived from arachidonic acid, and mediate a wide range of physiological functions, such as platelet aggregation, renin release, control of blood pressure, contraction of smooth muscle, and their well-recognized role in inflammation and immune response modulation. They are considered local hormones or autocoids because they act in a paracrine or autocrine fashion. Their activity is limited to the site of action by their short half-life and also by the fact that they are synthesized on demand and not stored in tissues[4,19].

Collectively with thromboxanes, they form a group of oxygenated fatty acid derivatives called prostanoids, usually derived from arachidonic acid. These oxygenated metabolites are also known as eicosanoids because they originate from a 20-carbon (eicosa) polyunsaturated acid[19].

### THE BIOSYNTHESIS OF PROSTAGLANDINS

Cyclooxygenase (COX) (the rate-limiting enzyme), also known as prostaglandin H synthase (PGH synthase), catalyzes the conversion of arachidonic acid (hydrolytically liberated from membrane phospholipids) to the PG endoperoxides, $PGG_2$ and $PGH_2$, with the addition of two oxygen molecules. In this pathway, two reaction steps can be differentiated, which may be catalyzed by different COX proteins. The first is the COX reaction in which the formation of a $C_5$ ring system occurs, leading to the formation of $PGG_2$, and the second is the peroxidase reaction in which the peroxide group at C-15 is reduced to an alcohol with the formation of $PGH_2$. $PGH_2$ is the precursor for the biologically active PGs and thromboxanes ($TXA_2$). Nonsteroidal anti-inflammatory drugs (NSAIDs) inhibit only the COX reaction of the PGH synthase[9,19].

### THE PROSTAGLANDIN PATHWAYS

Tissue damage activates phospholipase $A_2$ (PL-$A_2$), which causes arachidonic acid to be split off the cell membrane phospholipids. The release of arachidonic acid depends on which possible pathway it takes. There are two main pathways: the lipoxygenase pathway leads to the formation of leukotrienes and lipoxins, whereas the COX pathway leads to formation of PGs and thromboxanes[4,24]. PGs are local hormones that have specific effects on target cells close to their site of formation. They are rapidly degraded, so they are not transported to distal sites within the body. Examples include PGs, prostacycline, thromboxane, leukotrienes, and epoxyeicosatrienoic acids. They have roles in inflammation, fever, regulation of blood pressure, blood clotting, control of reproductive processes and tissue growth, and regulation of the sleep/wake cycle[4,13].

PGs and related compounds are collectively known as eicosanoids. They are produced from arachidonic acid, a 20-carbon polyunsaturated fatty acid (5,8,11,14-eicosatetraenoic acid)[19]. The fatty acid arachidonate is frequently esterified at the hydroxyl on carbon-2 of glycerophospholipids, especially phosphatidyl inositol, with arachidonate. Arachidonate is released from phospholipids by hydrolysis. Sites of hydrolytic cleavage by PL-$A_2$ and -C. PL-$A_2$ hydrolyzes the ester linkage between a fatty acid and the hydroxyl at carbon-2 of the glycerol backbone, releasing the fatty acid (e.g., arachidonate) and a lysophospholipid as products[13,19].

### PROSTAGLANDIN RECEPTORS

PGs and related compounds are transported out of the cells that synthesize them. They can affect other cells by interacting with plasma membrane G-protein–coupled receptors. Depending on the cell type, the activated G-protein may stimulate or inhibit formation of cyclic adenosine monophosphate (cAMP), or may activate a phosphatidyl inositol signal pathway leading to intracellular $Ca^{++}$ release[19].

### COX-1 AND COX-2 ISOFORMS

Two isoforms of $PGH_2$ synthase are designated COX-1 and COX-2. These two isoforms have similar primary protein structure (60% homology), size, substrate specificity, kinetics, and catalyze essentially the same reaction, but they vary in their expression and distribution. Structural analysis showed that the isoenzymes COX-1 and COX-2 had an amino acid sequence homology of approximately 60%. However, the isoforms encoded by different genes differ in their tissue distributions and regulation of expression. COX-1 is expressed constitutively in almost all cell types including thrombocytes and those present in kidney, stomach, and vascular endothelium, and is synthesized and regulated and called as a housekeeping enzyme that is involved in physiological adaptation[4,7,25]. COX-2, in contrast, is inducible and the induction can occur during tissue damage or inflammation in response to cytokines (tumor necrosis factor-α, interleukin-1), mitogens, and growth factors. The induction of COX-2 has been observed in macrophages and monocytes, endothelial cells, chondrocytes, and osteoblasts. An increased COX-2 level has also been registered in the synovial tissue of patients with rheumatoid arthritis (RA) and osteoarthritis (OA)[13]. These first findings led to the hypothesis that a selective blockade of the COX-2 isoform should lead to the inhibition of inflammation and pain without interfering with the COX-1–dependent effects in the gastrointestinal (GI) tissue, kidney, and in blood coagulation[24,25].

COX-1 is constitutively expressed at low levels in most cells throughout the body like the stomach, kidneys, platelets, and endothelial cells under normal physiologic conditions except red blood cells[13]. COX-1 is known to be essential for maintaining the integrity of the GI epithelium by inducing mucoprotective PGs, mainly $PGE_2$ and $PGI_2$, that have cytoprotective roles in the stomach[18]. Also, they are responsible for the production of housekeeping PGs to maintain the normal renal function of gastric mucosal integrity, vascular hemostasis, and the autocrine response to circulating hormones[24,25].

COX-2 is an inducible isoform that is found and expressed mainly in inflammatory and immune cells (neutrophils, macrophages, mast cells, etc). It is not only an inducible enzyme, but also it is unregulated 20-fold in macrophages, monocytes, synoviocytes, chondrocytes, fibroblasts, osteoblasts, and endothelial cells by various inflammatory stimuli. COX-2 is induced by proinflammatory substances and cytokines, such as interleukin-1 (IL1), tumor necrosis factor (TNF), lipopolysaccharides (LPS), mitogens (phorbolesters), reactive oxygen intermediates and chorionic gonadotropin in ovarian follicles, growth factors, platelet-derived growth factor (PDGF), transforming growth factor b (TGFb), epidermal growth factor, fibroblast growth factor (FGF), and the overexpression of COX-2 leads to increased expression of VEGF (vascular endothelial growth factor), which suggests that it may play an important role not only in the process of inflammation, but also in the control of cell growth[13]. At the site of inflammation, COX-2 is responsible for the generation of the hyperalgesic and proinflammatory PGs. COX-2 is also strongly expressed in human colon cancer cells and NSAIDs are thought to delay the progress of colon tumors possibly by causing apoptosis (programmed cell death) of the tumor cells[14]. In addition to being inducible, COX-2 is also present constitutively in the central nervous system, where it may be involved in fever and nerve transmission of pain. PGs catalyzed by COX-2 are also involved in ovulation and the birth process[4,24,25,30].

### PHYSIOLOGY AND PATHOPHYSIOLOGY OF PROSTAGLANDINS

PGs are formed in many types of cells within an organism. Their effects are complex and depend on the type of target cells. For this reason, it is difficult to generalize the physiological roles of individual PGs, because the same compound can sometimes exert opposite effects on different types of target cell. PGs are important in the regulation of thrombocyte aggregation, inflammatory processes, pain and fever induction, the regulation of vessel perfusion, and many other processes. NSAIDs function as antiinflammatory, antipyretic, and analgesic substances. According to the mechanism of action, the unwanted side effects of NSAIDs can also be explained (for example, erosion and bleeding in the GI tract, kidney function disorders, and disturbance of blood coagulation), which arise from a blockade of the physiological effects of $PGI_2$, $PGE_2$, and $TXA_2$. In this way, the ulcerogenic activity of NSAIDs can be derived from the physiological functions of $PGE_2$ and $PGI_2$. Both tissue hormones are cytoprotective towards the stomach and they stimulate the production of mucus and inhibit acid secretion. NSAIDs inhibit endogenous PG synthesis and this will remove the cytoprotective effects that lead to the promotion of ulcers[22,30,31].

### COX PRODUCTS

As previously explained, COX metabolizes arachidonic acid into $PGH_2$ intermediates and then $PGH_2$ is converted into biologically active products by the cell-specific enzymatic reactions. The products include not only classic PGs ($PGD_2$, $PGE_2$, and PGF2-alpha), but also $PGI_2$ and $TXA_2$[5,30].

$TXA_2$ is the predominate product of COX in platelets and macrophages, and is converted from $PGH_2$ by thromboxane synthase. The functional characteristics include platelet aggregation, vascular smooth muscle constriction, and bronchial smooth muscle constriction with a corresponding 30-sec tissue half-life[30].

$PGI_2$ is the major product of COX in microvascular endothelium, which is converted from $PGH_2$ by $PGI_2$ synthase. The functional characteristics include inhibition of platelet aggregation, inhibition of platelet and neutrophil adhesion, dilation of bronchial and vascular smooth muscle, and modulation of cholesterol efflux from arterial walls with a corresponding 3-min tissue half-life. Furthermore, the biosynthesis of $PGI_2$ is enhanced in the face of thrombogenesis and vasoconstriction to balance the physiologic milieu[30].

$PGD_2$ is a COX product that is found in mast cells and is formed by converting $PGH_2$ by endoperoxide-D isomerase. The functional characteristics include symptoms associated with histamine release (e.g., hypotension) and low-defined roles in immunologic processes. However, the formation of 9-alpha,11-beta-prostaglandin F (PGF) metabolites by preferential conversion of $PGD_2$ results in the inhibition of platelet aggregation and the contraction of vascular smooth muscle tissue. This might explain the occasional hypertensive patient observed with systemic mastocytosis[30].

$PGE_2$ is a significant product of COX in the gastric mucosa, renal medulla, microvascular endothelium, and in some tumors. This biologically active product is converted from $PGH_2$ by endoperoxide-E isomerase. In the gastric mucosa, $PGE_2$ preserves integrity by influencing mucus and bicarbonate secretion. It also maintains mucosal blood flow and participates in cellular repair. In the renal medulla, $PGE_2$ enhances vasodilatation and inhibits tubular sodium absorption and the deficiency of $PGE_2$, as observed in essential hypertension, may result in vasoconstriction. Furthermore, it may result in the stimulation of local osteoclasts, relaxation of bronchial smooth muscle tissue, contraction of uterine smooth muscle tissue, and modulation of presynaptic adrenergic neuron receptors[30,31].

### COX-2 INHIBITORS THERAPY

#### Gastrointestinal Safety with Cox-2 Inhibitors

Compared with the most commonly used NSAIDs, the COX-2 inhibitors have a better safety profile according to endoscopic erosions, fecal red blood cell loss, symptomatic ulcers, and ulcer complications. Endoscopy data from studies of 1,427 patients with OA showed that ulcers were significantly less common in the rofecoxib group than in the ibuprofen group at 24 weeks[31]. According to The *Medical Journal of Australia*, the COX-2 inhibitors do not eliminate the occurrence of ulcers or their serious complication, but they associated with considerably fewer peptic ulcers, upper GI symptoms, and bleeding than NSAIDs[26].

### COX-2 Inhibitors and Colon Cancer

Clinical trials with NSAIDs have demonstrated that NSAID treatment caused regression of pre-existing colon adenomas in patients with familial adenomatous polyposis[11,14]. Preclinical efficacy studies have provided evidence that several phytochemicals with anti-inflammatory properties and NSAIDs act to retard, block, or reverse colon carcinogenesis[14]. Equally exciting are opportunities for effective chemoprevention with selective COX-2 inhibitors including celecoxib and rofecoxib in a variety of preclinical models of colon cancer[11]. Cautions about Celebrex surfaced when the National Cancer Institute stopped using the drug for all participants in a large colorectal cancer prevention trial after an independent analysis found there was an increased risk of major fatal and nonfatal cardiovascular events for participants taking the drug compared with those taking a placebo[35]. COX-2 drugs are being used in more than 40 National Institutes of Health studies for the prevention and treatment of cancer, RA, OA, dementia, and other diseases, and now these agencies are currently re-evaluating their use[35].

### Pain Relief

Rofecoxib (50 mg) has been shown to be superior to placebo and equivalent to naproxen sodium (550 mg) in the 12 h after being taken for orthopedic surgical pain relief and for dysmenorrhea and equivalent to ibuprofen (400 mg) after third-molar tooth extraction (E2). Celecoxib in a dose of 100 or 200 mg was significantly better than placebo for pain after third-molar extraction and no different than ibuprofen (400 mg) or naproxen sodium (550 mg)[15].

### Osteoarthritis

In a 12-week trial of more than 1,000 patients comparing 50, 100, and 200 mg of celecoxib twice daily with 500 mg of naproxen twice daily or placebo, the 100- and 200-mg doses of celecoxib were as effective as the naproxen. Although 50 mg of celecoxib twice daily was better than placebo, it was not as effective as the higher doses. Rofecoxib in doses of 12.5 and 25 mg once daily has been shown to be significantly better than placebo, as effective as 2.4 g of ibuprofen daily (over 6 weeks), and as effective as 150 mg of diclofenac daily (over 1 year) for OA of the knee[10,15].

### Rheumatoid Arthritis

A 3-month, double-blind, placebo-controlled study comparing 500 mg of naproxen twice daily, placebo, and celecoxib in doses of 100, 200, or 400 mg twice daily in more than 1,100 patients with RA showed that all celecoxib doses and naproxen were effective for pain and inflammation throughout the 12 weeks[10,15].

### Other Benefit of Using COX-2 Inhibitor

Long-term administration of a selective COX-2 inhibitor also significantly decreased proteinuria and reduced extracellular matrix deposition, as indicated by decreases in immunoreactive fibronectin expression and mesangial matrix expansion. In addition, COX-2 inhibition reduced expression of TGF-

beta, plasminogen activator inhibitor-1, and vascular endothelial growth factor in the kidneys of diabetic hypertensive animals.

### ADVERSE EFFECTS

COX-2 inhibiting drugs were developed to provide the anti-inflammatory effects of NSAIDs with a reduced risk of GI bleeding. Concerns have found that COX-2 inhibitors could have cardiovascular and renal adverse effects similar to those of nonselective NSAIDs, such as raised systemic vascular resistance and decreased renal perfusion[17]. The *New England Journal of Medicine* reports that anti-inflammatory drugs (prescription and over-the-counter, which include ibuprofen, naproxen, aspirin, and over 20 others) alone cause over 16,500 deaths and over 103,000 hospitalizations per year in the U.S.[22].

#### Hematological Effects

Hematological side effects of traditional NSAIDs are rare. Agranulocytosis (i.e., decreased platelet aggregation; prolonged bleeding time) and aplastic anemia is the most serious. Phenylbutazone and indomethacin are the NSAIDs most commonly associated with agranulocytosis[29].

#### Cardiovascular Effects

The new cardiovascular warning for valdecoxib (Bextra) was issued when results from a recently completed study by Pfizer, the drug's manufacturer, revealed that heart attacks, strokes, deep vein thrombosis, and pulmonary embolisms occurred more often among patients taking the drug[26,31]. Rofecoxib (Vioxx) was pulled from the market in September 2004 because it increased cardiovascular risk among patients who took the drug for at least 18 months[22,31].

#### Central Nervous System Effects

NSAIDs can cause headaches, confusion, dizziness, drowsiness, tremors, aseptic meningitis, vertigo, tinnitus, neuropathy, behavior disturbance, toxic amblyopia, and transient transparent corneal deposits, and aggravate psychiatric illness like epilepsy and Parkinsonism. Hallucinations, depression, tremors, drowsiness, and ingestion of indomethacin are associated with the highest incidence of NSAID-associated CNS symptoms. The presence of the COX-2 protein in neuronal and glial cells following ischemic injury has been reported, suggesting a possible pathologic role for COX-2. No consistent association is found between COX-2 expression and neurologic abnormalities[29].

#### Gastrointestinal Effects

GI side effects associated with NSAID therapy are common. Approximately 30–60% of NSAID users experience some abdominal discomfort or dyspepsia. These symptoms do not necessarily correlate with endoscopic findings. Approximately 40% of persons with erosive gastritis are asymptomatic, while almost 50% of patients with dyspepsia have no evidence of mucosal damage on endoscopy.

Endoscopic studies of arthritic patients receiving chronic NSAID therapy revealed that 50–75% of subjects have gastroduodenal lesions ranging from small subepithelial hemorrhages and mucosal erosions to ulcers. Studies showed the prevalence of peptic ulcers in patients on chronic NSAIDs to be about 25%. Gastric ulcers were seen in 15% of patients, while duodenal ulcers were present in 10%. Lower GI

complications are not as common as those in the upper GI tract, but they are potentially serious. NSAID use can be associated with colonic ulceration, perforation, inflammation, strictures, and diarrhea. Patients with inflammatory bowel disease could suffer an exacerbation of their disease by taking NSAIDs. Also, it may cause esophagitis and esophageal strictures, nausea, vomiting, dyspepsia, diarrhea, constipation, gastric mucosal irritation, erosions, gastric ulcerations, fecal blood wasting, GI hemorrhage, small and large bowel erosive disease, and small and large bowel strictures[29].

### Hepatic Effects

Liver injury from NSAIDs ranges from asymptomatic transaminase elevation to clinical hepatitis. Fatal liver damage may rarely occur. Liver function tests are advised at the beginning of treatment and after 3 months because side effects frequently appear during the initial 3 months of therapy. NSAIDs should be discontinued when alanine transferase (ALT) and asparate transferase (AST) are elevated to be two to three times the normal levels. A recent review identified the three NSAIDs most commonly associated with hepatic toxicity as sulindac, diclofenac, and aspirin[29].

### Coagulation Effects

There are several mechanisms by which traditional NSAIDs increase the risk and severity of bleeding, especially in a patient already taking warfarin. A direct hypoprothrombinemic effect occurs by depressing the vitamin K–dependent synthesis of clotting factors VII, IX, and X. Also, NSAIDs displace warfarin from plasma albumin, which is a transient effect, since there is an increase in the clearance of unbound warfarin until the previous concentration is again reached. Because platelets contain COX-1 and not COX-2, traditional NSAIDs demonstrate antiplatelet effects by inhibiting COX-1. COX-2 selective agents do not interfere with platelet activity. Celecoxib administered at doses of 600 mg bid does not interfere with serum thromboxane, bleeding time, or platelet aggregation as is seen with naproxen. Moreover, celecoxib, when combined with 2–5 mg/day of warfarin, does not prolong the prothrombin time. A role for celecoxib as prophylaxis for myocardial infarction and stroke is not likely, due to its lack of antiplatelet activity[29,31,35].

### Cartilage Effects

PGs are well-documented modulators of articular cartilage metabolism and bone resorption. According to some reports, NSAIDs can exacerbate cartilage erosion, produce bony destruction of the femoral head in OA patients, and accelerate the progression of joint damage. COX-2 was recently shown to play a role in the bone loss induced by IL1. An increase in COX-2 production in cartilage explants suggests it may have a role in cartilage repair[29]. Celecoxib and rofecoxib, commonly prescribed to reduce pain and inflammation caused by arthritis, may also impair healing of bone fractures as suggested by an animal study[15].

### Pulmonary Effects

A 4- to 14-fold increase in COX-2 levels in asthmatic subjects has been reported, suggesting a possible COX-2 role in the pathogenesis of asthma. COX-2 blockade may be therapeutic in asthma, whereas older NSAIDs can cause bronchoconstriction and edema, especially in aspirin-sensitive patients[29,31].

### Reproductive Tract Effects

The COX-2 enzyme is apparently induced in ovulation. Studies with COX-2–null mice (mice deficient in COX-2) have documented reproductive failures at ovulation, fertilization, and implantation. Inhibition of COX-2 by NSAIDs may explain the infertility secondary to delayed or blocked follicular rupture associated with their use. COX-2 expression is prominent in the human amnion at term by 100-fold compared with earlier in gestation, which suggests a possible role for COX-2 inhibiting preterm labor. COX-2 inhibitors are contraindicated at or near term. Recent data showed that the fetus produces $PGF_{2a}$-inducing luteolysis, leading to uterine contractions. This process reduces maternal progesterone levels and induction of oxytocin receptors in the myometrium leading to parturition[32]. COX-2 also influences fertilization and implantation. This enzyme is required for normal oocyte development and probably for generation of the enzymes necessary to rupture the follicle. Following fertilization, COX-1 prepares the wall for interaction with the embryo, while COX-2 and prostaglandin receptors mediate implantation[29,32]. Also, indomethacin, which inhibits both COX-1 and COX-2, is effective for delaying premature labor, but its use is limited by serious complications to the fetus and neonate, including adverse effects on the ductus arteriosus (DA). Chronic inhibition of COX-2 during pregnancy (gestation days 15–18) significantly increased neonatal mortality by preventing closure of the DA after birth, whereas acute COX-2 inhibition near the end of term (gestation day 18) constricted the fetal DA. In contrast, the inhibition of COX-1 during pregnancy lacked these prenatal and postnatal adverse effects on the DA and effectively delayed the initiation of full-term labor and LPS-induced preterm labor[32].

### Renal Effects

Effects include reversible acute renal failure, chronic renal failure and interstitial fibrosis, nephrotic syndrome, interstitial nephritis, fall in Glomerular Filtration Rate and inhibition of renin release, alterations in tubular function and fluid-electrolyte disturbances, papillary necrosis, uric acid stones, glomerulopathy, and interference with diuretic induced natriuresis[26,29]. COX-2 and the renal side effects will be demonstrated briefly in this research.

### Hypersensitivity

Hypersensitivity reactions include exacerbation of asthma, rashes, urticaria, and photosensitivity. The skin reactions like Stevens-Johnson syndrome and toxic epidermal necrolysis are most likely to occur in the first 2 weeks of treatment with NSAIDs[29]. In addition, although other COX-2 inhibitors and traditional NSAIDs also carry a risk of serious skin reactions, the rate among valdecoxib users appeared higher, according to the FDA[26,33].

## THE FUNCTIONAL ROLES OF COX-2 IN THE KIDNEYS

PGs play an integral role in renal physiology, renal microvascular homodynamics, renin release, and tubular salt and water reabsorption[13,31]. These agents can be particularly harmful to renal function in patients depending on the vasodilatory actions of PGs in the kidney. PGs are necessary to preserve renal hemodynamics in conditions associated with high adrenergic and renin-angiotensin stimulation, such as heart failure, liver failure, and renal insufficiency[25,27]. Localization studies show that COX-2 is expressed in the adult rat renal cortex in a restricted subpopulation of cells in the cortical thick ascending limb (c/TAL) cells in the region of macula densa (MD) (a group of cells in the wall of the distal renal tubule next to the juxaglomerular cells, that are sensitive to changes in the salt concentration of the filtrate in the tubule) and in the medullary interstitial cells. COX-2 is also localized to the vasa recta of the medulla. The renal localization is consistent with a role for COX-2 in regulating glomerular function, blood pressure, and sodium excretion[13,25].

As previously discussed, these isoforms are expressed in distinct, but important, areas of the kidney. COX-1 predominates in vascular smooth muscle and collecting ducts, whereas COX-2 predominates in the MD and nearby cells in the c/TAL. COX-2 is also highly expressed in medullary interstitial cells. Whereas COX-1 expression does not exhibit dynamic regulation, COX-2 expression is subject to regulation by several environmental conditions, including salt intake, water intake, medullary tonicity, growth factors, cytokines, and adrenal steroids[13,28]. $PGI_2$ and $PGE_2$ maintain renal blood flow in states of effective volume depletion such as congestive heart failure (CHF), liver cirrhosis, and true volume depletion seen with chronic diuretic therapy. Also, $PGI_2$ and $PGE_2$ preserve renal blood flow by antagonizing the vasoconstrictive effects of angiotensin-II (Ag-II) and norepinephrine. Because of these properties, NSAID use may be associated with peripheral edema, hyperkalemia, acute renal failure in patients with hypovolemia, altered intrarenal plasma flow, nephrotic syndrome with interstitial nephritis, and papillary necrosis[13,3].

Most, but not all, experimental studies have supported a role for COX-2 in MD mediation of renin release. In isolated, perfused, glomerular preparations, renin release induced by MD perfusion with a low chloride solution was inhibited by a COX-2 inhibitor, but not a COX-1 inhibitor. *In vivo* studies in rats indicated that increased renin release in response to low-salt diet, ACEI, loop diuretics, or aortic coarctation could be inhibited by administration of COX-2–selective inhibitors[13].

Randomized crossover studies in healthy humans who were administered furosemide and/or low-sodium diet have also demonstrated inhibition of renin release by a COX-2 inhibitor like rofecoxib[13]. Also, in healthy human subjects on normal diets, COX-2 inhibitors have minimal effects on renal hemodynamics. However, COX-2 inhibitors decrease GFR in salt-depleted subjects or the elderly. Therefore, COX-2 expression in the renal cortex seems to be inhibited by Ag-II and stimulated under conditions of low-sodium intake or diuretic administration. If the activity of the renin-angiotensin system is diminished or insufficient to maintain electrolyte balance, then, at least in some circumstances, increased COX-2 expression and synthesis of PGs activate expression and release of renin leading to increased activity of Ag-II and aldosterone and resulting in increased tubule reabsorption, thereby facilitating re-establishment of intravascular volume homeostasis. Once this is achieved, the expression of COX-2 is reduced through inhibition by Ag-II, decreasing a stimulus for renin production and release. Thus, PGs facilitate the TGF response to low-salt delivery to MD by increasing Ag-II levels and by preventing Ag-II from decreasing GFR. COX-2 may be involved in maintaining sodium excretion, GFR, and renal blood flow, especially in elderly and/or patients with decreased circulating volume. In states of relative or absolute intravascular volume depletion, COX-2 inhibitors may exert deleterious effects on renal function[13]. Both COX-1 and COX-2 are expressed in the renal medulla. Interestingly, the COX-1 and COX-2 isoforms are differentially localized in the renal medulla, as demonstrated in rats, rabbits, and humans, with COX-1 expressed primarily in the collecting duct and COX-2 predominantly in interstitial cells. Human nephrectomy and autopsy specimens have shown COX-2 at glomerular podocytes, afferent arterioles, and the endothelial cells of arteries and veins (suggesting a role in renal hemodynamics), and at the MD, thick loops of Henle, and medullary interstitial cells (implying a role in salt and water balance)[20,25].

### COX-2 and Vasodilator Action

Studies of prostanoid-dependent control of renal blood flow and GFR by the MD indicate that vasodilator and vasoconstrictor prostanoids may contribute to regulation of TGF. Vasodilatory PGs appear to maintain renal blood flow and GFR in the face of vasoconstrictors, such as Ag-II or norepinephrine, by blunting constriction of the afferent arteriole. Some studies suggest that some COX-2–derived prostanoids are predominantly vasodilators like $PGE_2$ in the adjacent afferent arterioles and using selective COX-2 inhibitors may contribute to the decrease in GFR[13].

### Renal Medullary COX-2

Medullary $PGE_2$ plays an important role in regulating salt and water reabsorption in the medullary thick ascending limb and collecting duct. Sodium retention is a well-recognized side effect of nonselective NSAIDs because COX-1 is abundantly and constitutively expressed in cortical and medullary collecting duct and it has been hypothesized to be involved in the natriuretic response. In this regard, acute increase in renal interstitial hydrostatic pressure by direct renal interstitial volume expansion is known to increase sodium excretion and infusion of nonselective NSAIDs, but not COX-2 inhibitors, will blunt this natriuretic response. Furthermore, in a rat model of cirrhosis and ascites, a COX-1 selective inhibitor (but not a COX-2 selective inhibitor) decreased sodium excretion and impaired the diuretic and natriuretic responses to furosemide. However, COX-2 also appears to be involved in modulating sodium excretion. Salt loading downregulates COX-2 expression in renal cortex, but upregulates its expression in renal medulla. The increased COX-2 may mediate natriuresis. COX-2 inhibitors have also been shown to cause sodium retention in a small percentage of human subjects without renal impairment, and decreased urinary sodium excretion for the first 72 h of administration of COX-2 inhibitors is seen in subjects with salt depletion or in elderly subjects on a normal salt diet. When renal cortical blood flow (CBF) and medullary blood flow (MBF) were selectively measured in mice, it was found that acute infusion of a COX-1 selective inhibitor did not affect either CBF or MBF. In contrast, a COX-2 selective inhibitor significantly reduced MBF without altering CBF[1,13,25].

The vasoconstriction associated with adrenergic and angiotensin activation increases intrarenal release of $PGE_2$ and $PGI_2$. Renal blood flow and GFR are maintained by the vasodilatory effect of these PGs. In addition to their homodynamic effects, PGs are necessary to sustain salt and water excretion. For example, $PGE_2$ inhibits reabsorption of sodium and chloride and antagonizes antidiuretic hormone[1,13,25,27].

As vasodilators, PGs allow increased blood flow to the kidneys, which helps more water to be excreted through urine. The danger of fluid overload is greater with a nonsteroidal present because it is harder to dilute the urine when there are no normal COX-2 PGs that are supposed to be there[13,31,36].

In healthy and nonsodium-depleted adults, COX-2 activity more specifically regulates renal sodium and water balance than GFR and COX-2 inhibition in predisposed individuals, such as those with age-related decreases in GFR or sodium depletion, and may deteriorate renal function[13,27].

### COX-2 INHIBITORS AND THE KIDNEY

In the kidney, PGs are important mediators of vascular tone, salt and water balance, and renin release. COX-2 is constitutively expressed in the kidney and is highly regulated in response to alterations in intravascular volume. COX-2 metabolites have been implicated in maintenance of renal blood flow, mediation of renin release, and regulation of sodium excretion. COX-2 inhibitors may transiently decrease urine sodium excretion in some subjects and induce mild to moderate elevation of blood pressure. Furthermore, in conditions of relative intravascular volume depletion and/or renal hypoperfusion, the interference with COX-2 activity can have deleterious effects on maintenance of renal blood flow and GFR. In addition to physiological regulation of COX-2 expression in the kidney, increased renal cortical COX-2 expression is seen in experimental models associated with altered renal homodynamic and progressive renal injury (decreased renal mass, poorly controlled diabetes), and long-term treatment with selective COX-2 inhibitors ameliorates functional and structural renal damage in these conditions[12,13,23,25].

It was hypothesized that the renal effects of NSAIDs might be linked to COX-1 inhibition. With the advent of selective COX-2 inhibitors, increasing experimental and clinical evidence has also indicated important roles for COX-2 metabolites in physiological and pathophysiologic modulation of renal function[20,25].

The present observations that COX-2 inhibition blunts Ag-II–induced natriuresis suggest that COX-2 generates PGs that promote renal salt excretion. It is well established that treatment of in vitro microperfused medullary thick ascending limbs or collecting ducts with basolateral PGE2 inhibits salt absorption in these segments. The demonstration that COX-2 is expressed in medullary interstitial cells adjacent to these segments, coupled with the observation that Ag-II stimulates renal medullary synthesis of PGE2 and PGI2 by COX-2, is consistent with a model whereby Ag-II promotes natriuresis, in part, via a paracrine effect of PGE2 inhibiting salt absorption by collecting ducts and thick ascending limbs. By blocking PGE2 and PGI2 synthesis, COX-2 inhibitors simultaneously enhance epithelial salt absorption and further constrict vascular resistance beds. These events could result in hypertension and edema in humans[1,13,25,34].

### CAUTIONS ABOUT NSAIDS AND COX-2 INHIBITORS

There are no significant differences from conventional NSAIDs and the selective one. Therefore, similar precautions and contraindications apply to both drug groups. An absolute contraindication for their use is the presence of hyperkalemia. Caution is required for those patients with renal risk factors, which include those over 60 years of age, GFR $\leq$ 60 ml/min, patients on salt-restricted diets, those receiving diuretics, angiotensin-converting enzyme inhibitors, Ag-II receptor blockers, cyclosporin, aspirin, and patients with cirrhosis or CHF. These patients are at a higher risk of deterioration of renal function if NSAIDs and COX-2 inhibitors therapy is prescribed. Also plasma sodium, potassium, and creatinine levels, blood pressure, the presence of edema, and urinalysis should be monitored in those cases[26]. The manufacturers of COX-2 inhibitors caution against the administration of these agents in patients with pre-existing renal disease and considerable dehydration. In addition, growing evidence suggests that COX-2 inhibitors should be prescribed with caution or not at all for patients in whom traditional NSAIDs pose a risk for renal failure, including those with heart failure, sodium depletion, and pre-existing hepatic or renal insufficiency[25,36].

### RENAL EFFECTS AND COX-2 INHIBITORS STUDIES

The COX-2 inhibitors are shown to decrease renal prostacyclin production under physiological conditions in young volunteers. In the same population, COX-2 inhibitors caused a transient retention of sodium, but no depression of the GFR. In a different study of 75 elderly patients (aged 60–80 years) on a low-sodium diet, low and high dosages of rofecoxib were compared with indomethacin and placebo, and decreases in the GFR were comparable in the rofecoxib- and indomethacin-treated groups . Another recent study of celecoxib found similar renal effects. Several studies have demonstrated that the effects of COX-2 inhibitors on renal function are expected to be similar to those observed with nonselective NSAIDs[13].

Several large randomized clinical trials have reported the frequency of renal adverse effects of selective COX-2 inhibitors. A study comparing rofecoxib and naproxen in more than 8,000 patients with RA reported that the frequency of renal adverse effects was similar between the groups (1.2 and 0.9% in the rofecoxib and naproxen groups, respectively). The Celecoxib Long-Term Arthritis Safety Study (CLASS) reported a 5% frequency of renal effects (peripheral edema, hypertension, and increased serum creatinine) for the celecoxib group and 6.6% for the NSAIDs group. In this study, 0.9% of patients in the celecoxib group and 1.5% of patients in the NSAIDs group had increases in serum creatinine of 1.8 mg/dl or more, increases in serum urea nitrogen of 40 mg/dl or more, or both. Although the frequency of renal side effects with selective COX-2 inhibitors in these trials was low, it is important to note that patients with impaired renal or hepatic function or volume depletion were excluded from participating. More recently, a study using the World Health Organization/Uppsala Monitoring Centre Safety Database concluded that the adverse renal effects of celecoxib appear to be similar to those of traditional NSAIDs[13,27].

The effects of COX-1 and COX-2 inhibition on renal homodynamic and water balance were examined in 36 healthy elderly volunteers (age range 59–80 years). Individuals who had creatinine clearances less than 50 ml/min, serum creatinine levels higher than 2 mg/dl, or who required pharmacologic therapy for hypertension or diabetes were excluded from the study. Participants adhered to a diet that limited sodium to 200 mEq/day; those who maintained sodium balance (based on weight and 24-h urine collection) were enrolled. Volunteers were randomized to receive rofecoxib 50 mg/day, indomethacin 50 mg three times a day, or placebo. After 2 weeks of therapy, their GFRs and creatinine clearances decreased significantly from baseline in the indomethacin group, but no differences were found in the rofecoxib and placebo groups. Urinary 6-keto-$PGF_1$, an index of the renal biosynthesis of prostacyclin, was significantly inhibited by indomethacin and rofecoxib, but was not affected by placebo. Urinary sodium excretion was significantly decreased from baseline during the first 72 h of treatment in the rofecoxib and indomethacin groups. This effect was not significantly different between the treatment groups. At day 14, there was no difference in urinary sodium excretion between participants receiving indomethacin, rofecoxib, or placebo. Selective COX-2 inhibition decreased renal prostacyclin synthesis to the same extent as nonselective COX-1 and COX-2 inhibition and transiently decreased sodium excretion independent of renal homodynamic[1,13,27].

### COX-2 AND THE CARDIOVASCULAR SYSTEM

Studies and research have demonstrated beneficial cardiac effects of the COX-2 enzyme and potential harmful effects of COX-2 inhibitors. It has been demonstrated that COX-2 mediates the cardioprotective effects of the late phase of ischemic preconditioning by examining the late phase of ischemic preconditioning in rabbits. They demonstrated that ischemic preconditioning resulted in a rapid increase in myocardial COX-2 mRNA levels. This was followed by an increase in COX-2 protein expression and in the myocardial content of $PGE_2$ and 6-keto-PGF (1-alpha). Administration of two unrelated COX-2 selective inhibitors (NS-398 and celecoxib) 24 h after ischemic preconditioning abolished the ischemic preconditioning-induced increase in tissue levels of $PGE_2$ and 6-keto-PGF (1-alpha)[6,21].

The same doses of NS-398 and celecoxib, given 24 h after ischemic preconditioning, completely blocked the cardioprotective effects of late preconditioning against both myocardial stunning and myocardial infarction, indicating that COX-2 activity is necessary for ischemic preconditioning to occur. These results demonstrate that upregulation of COX-2 plays an essential role in the cardioprotection afforded by the late phase of ischemic preconditioning[5,21]. The important role of COX-2 in the homeostatic balance, by showing that the increase in time to vascular occlusion with aspirin in a canine coronary thrombosis model, was abolished with a selective COX-2 inhibitor, celecoxcib. In the study of canine circumflex coronary artery thrombosis, oral high-dose aspirin produced a significant increase in time to arterial occlusion[16]. This observed increase in time to occlusion was abolished when celecoxib was coadministered to animals dosed with aspirin. The study also suggests the important role of COX-2–derived $PGI_2$, and it raises concerns regarding an increased risk of acute vascular thrombotic events in patients receiving COX-2 inhibitors[5,8].

### COX-2 INHIBITORS AND CARDIOVASCULAR EFFECTS

COX-2 inhibitors were developed to reduce the GI toxicity of conventional NSAIDs agents. However, COX-2 inhibitors decrease vascular $PGI_2$ production and may disrupt the normal homeostatic balance, leading to a prothrombotic state by decreasing vasodilatory and antiplatelet aggregatory $PGI_2$ production. Also, COX-2 inhibitors may affect the balance of prothrombotic eicosanoids. $TXA_2$ may lead to increased cardiovascular thrombotic events[5,8,31].

Blood pressure may rise, sometimes substantially, with prescribing of selective COX-2 inhibitors[17,38]. NSAIDs have increased the blood pressure of subjects in several trials by inhibiting PG

synthesis and they interfere with systemic and renal vasodilatation, glomerulo-filtration, tubular secretion of fluids and electrolytes, adrenergic neurotransmission, and the renin-angiotensin-aldosterone system, leading to hypertension[1,5,8].

A recent meta-analysis of 50 studies reported that NSAIDs increased the mean blood pressure by approximately 5 mmHg. Investigators also found that hypertensive patients taking blood pressure–lowering drugs were more severely affected by NSAIDs use than normotensive volunteers given antihypertensives. The hypertensive effect of NSAIDs appears to be most common in patients taking angiotensin-converting enzyme inhibitors, diuretics, and beta-blockers. However, the antihypertensive effect of calcium channel blockers does not appear to be affected by NSAIDs[1,5,31].

Blood pressure should be monitored and the dose of antihypertensive medication adjusted to maintain blood pressure at treatment target levels. Selective COX-2 inhibitors can exacerbate cardiac failure and occasional patients may present with pulmonary edema. There is evidence that RA may be a risk factor for cardiovascular disease. A large, long-term clinical trial in RA, using supratherapeutic doses of rofecoxib, showed an increase in the rate of myocardial infarction compared with naproxen. So, caution is important when considering the use of selective COX-2 inhibitors in patients with risk factors for coronary disease, especially in those with RA[18,26].

### COX-2 Inhibitors and Thrombosis

Two vasoactive PGs balance prothrombotic and antithrombotic forces in the body. $PGI_2$, which is synthesized by endothelial cells primarily via a COX-2-dependent pathway, dilates blood vessels, inhibits platelet aggregation by elevating platelet cAMP, induces vasodilation, and prevents the proliferation of vascular smooth muscle cells *in vitro* and these series of effects collectively decrease the propensity to thrombosis. On the other hand, $TXA_2$ (formed by COX-1–dependent pathway) presents in platelets and enhances platelet aggregation, vasoconstriction, and vascular proliferation in response to injury and thereby increasing the likelihood of thrombosis. Also, $PGI_2$ is the main source of a fat that protects the heart from factors that activate the clotting system, harden the arteries, and raise blood pressure[1,5,7]. The delicate balance between platelet-induced $TXA_2$ and $PGI_2$ maintains normal blood flow and modulates thrombogenic responses to injury. COX-2 seems to play a pivotal role in the production of endothelial prostacyclin ($PGI_2$)[21]. Conversely, platelets produce $TXA_2$ via the action of COX-1. A balancing of prothrombotic and antithrombotic effects can be proposed during NSAID administration as they inhibit both COX-1 and COX-2 and the potential imbalance induced by COXIBs (inhibiting endothelial $PGI_2$ synthesis, but not platelet $TXA_2$ synthesis) may lead to increased risk of thrombosis[7,31,34].

Valdecoxib elevates the incidence of heart attack and stroke threefold in coronary artery bypass graft (CABG) surgery patients[34]. Also, adding the COX-2 inhibitors not only failed to add to the beneficial effects of the $TXA_2$ blocker, but also may have caused disturbing changes in the makeup of the atherosclerotic plaques that would result in a loss of stability of the plaque, making it more likely to rupture and activate the clotting that leads to heart attack or stroke[1,31,34,35].

A new study in mice adds to the concern that COX-2 inhibitors may increase the risk of heart attacks in people with heart disease. One of the COX enzymes (COX-1) makes thromboxane, which causes blood vessels to constrict and platelets to become sticky and these steps are important in a heart attack or stroke. Also, COX-2 enzyme is a major source of prostacyclin, a cardioprotective protein, which dilates blood vessels and prevents platelets from clumping together. In a healthy person, the two COX enzymes are thought to balance each other so blood does not clot excessively[5].

NSAIDs and aspirin inhibit the formation of platelet-derived thromboxane and endothelial prostacyclin and COX-2 inhibitors preferentially suppress the vasodilator and platelet-inhibitory PGs without blocking the vasoconstrictive and platelet-activating PGs, which could result in a prothrombotic effect. These effects may differ among structurally distinct COX-2 inhibitors with different levels of COX-1 or COX-2 selectivity[18,35]. Low-dose aspirin, known to have an antithrombotic effect, largely inactivates the platelet COX-1, thereby reducing only the levels of $TXA_2$ and decreasing blood clotting, but selective inhibition of COX-2 prevents the synthesis of $PGI_2$, leaving the prothrombotic $TXA_2$ unopposed[5,21].

Two randomized multi-center clinical trials with COX-2 inhibitors were conducted. These studies also looked at myocardial infarction rates in the placebo group (23,407 patients) in a meta-analysis of four large aspirin studies. They found that the annual myocardial infarction rate in the aspirin placebo group was 0.52%. This compared with 0.74% for the COX-2 inhibitor rofecoxib in the Vioxx Gastrointestinal Outcomes Research (VIGOR) study and 0.80% for the inhibitor celecoxib in the CLASS study[5]. Aspirin use was not permitted in the VIGOR study, in which 8,076 patients were randomized to receive rofecoxib 50 mg/day or naproxen 1,000 mg/day. There were 111 cardiovascular events in the rofecoxib arm and 50 events in the naproxen arm. However, aspirin use was permitted in the CLASS study, in which 8,059 patients were randomized to 400 mg of celecoxib twice a day, 800 mg of ibuprofen three times a day, or 75 mg of diclofenac twice a day. The result could be an increase in congestive cardiac failure in susceptible patients. Mamdani[17] and colleagues used databases on prescription drugs and hospital admissions in a Canadian province to look for any association between COX-2 inhibitor use and hospital admission for congestive cardiac failure. From a total population of 1.3 million persons aged 65 years or older, the investigators identified patients who were prescribed COX-2 inhibitors (rofecoxib and celecoxib) or nonselective NSAIDs from April 2000 through March 2001. Using the databases, they identified 14,583 NSAID-naive persons who started rofecoxib therapy and 18,908 NSAID-naive persons who started celecoxib therapy. There were 5,391 first-time users of nonselective NSAIDs (predominately diclofenac plus misoprostol, naproxen, or ibuprofen). These groups of patients were compared for admission to hospital because of heart failure with 100,000 control patients who represented the non-NSAID users in the population age group. The two groups using COX-2 inhibitors were comparable. For example, compared with the NSAID users and control subjects, the patients using COX-2 inhibitors were more likely to be female, to have had echocardiography, and to have received loop diuretics, digoxin, and several cardiac drugs, including beta blockers, calcium channel antagonists, and angiotensin-converting enzyme inhibitors. During the study, 654 patients were admitted to a hospital for treatment of CHF. The rate of admission was significantly increased for those receiving rofecoxib and NSAID compared with the control group. The risk of hospital admission was significantly increased in those receiving rofecoxib compared with those receiving NSAIDs. These results are consistent with studies showing more marked changes in blood pressure and edema with rofecoxib and could be related to the longer half-life and other pharmacokinetic properties of this drug[16,17].

### COX-2 Inhibitors and Hypertension

NSAIDs may elevate blood pressure and antagonize the blood pressure–lowering effect of antihypertensive medication to an extent that may potentially increase hypertension-related morbidity. COX-2 inhibitors may also affect blood pressure. Rofecoxib significantly elevated systolic blood pressure (SBP) in rats with normal-salt or high-salt diet, but not in rats on low-salt intake, which suggested that the hypertension induced by COX-2 inhibition can occur independently of a genetic predisposition to hypertension and can be prevented by salt deprivation[2,3,5,7].

The Successive Celecoxib Efficacy and Safety Studies (SUCCESS) VI and VII compared the renal safety in older hypertensive OA patients (890 and 1,092 patients in VI or VII, respectively) and found that at week 6, rofecoxib was more likely to increase the SBP than celecoxib. Rofecoxib caused the greatest increase in SBP in patients receiving ACEIs or beta-blockers, whereas those on calcium channel antagonists or diuretic monotherapy receiving either celecoxib or rofecoxib showed no significant increases in blood pressure. However, in a randomized, double-blind, placebo-controlled, parallel-group, clinical trial involving 178 patients with essential hypertension, using 24-h ambulatory recordings, high doses (400 mg/day, twice the recommended dose) of celecoxib did not show significant alteration of the antihypertensive effect of the ACEI, lisinopril during a 4-week period. The effects of most antihypertensive agents, except calcium channel blockers and Ag-II receptor antagonists, require the synthesis of vasodilator PGs. Recent studies have suggested that at least one COX-2 inhibitor, rofecoxib, appears to interfere with antihypertensive effects of ACEIs and beta-blockers, but not calcium channel blockers[5].

#### *COX-2 Inhibitors and Platelet Aggregation*

As previously mentioned, COX-1 enzymes cause platelet aggregation, which means that clotting is inhibited without them. In contrast, COX-2 enzymes are vasodilators. The blockage for both COX-1 and COX-2 by traditional NSAIDs create a balancing state. A nonselective agent helps to keep platelets from sticking, while at the same time blocking vasodilation. The smaller vessel is not a problem, because the platelets are not sticking. But when the patients use COX-2 inhibitors, there is no inhibiting for COX-1 enzymes and this will lead to vasoconstriction without platelet aggregation inhibition[7,31].

Studies used mice with an absent PG receptor that were hypnotized so that they should mimic the clinical effect of taking COX-2 agents, as these drugs would inhibit PG production without affecting $TXA_2$. The COX-2 knockout resulted in an enhanced proliferative response to injury and in a significant increase in $TXA_2$ biosynthesis. These results suggest that $PGI_2$ may modulate the platelet-vascular interactions *in vivo*, and that $PGI_2$ may have a beneficial effect by specifically limiting the prothrombotic response to $TXA_2$[7,21].

### CONCLUSION

The complexity and fundamental role of the prostanoid network in the kidney and the heart make it extremely difficult to interfere with this system safely. Risks range from modest, but significant, variations in blood pressure that may affect cardiovascular mortality in the long to short term, to life-threatening conditions such as acute renal failure and many other potentially serious side effects and disorders. Considering the physiological role of COX-2 in the kidney and heart and the few but serious reports of side effects, COXIBs may not be as safe for the kidneys as initially hoped. Until specific clinical trials and analyses of adverse event reports on sufficiently large numbers of patients clarify the renal risk and cardiovascular profile of these new agents, nephrologists and cardiologists should consider COXIBs equivalent to NSAIDs. More work is warranted to refine and test different strategies.

## References

1. Qi, Z., Hao, C.-M., Langenbach, R.I., Breyer, R.M., Redha, R., Morrow, J.D., and Breyer, M.D. (2002) Opposite effects of cyclooxygenase-1 and -2 activity on the pressor response to angiotensin II. *J. Clin. Invest*. **110**, 61–69.
2. Francois, H. and Coffman, T.M. (2004) Prostanoids and blood pressure: which way is up? *J. Clin. Invest*. **114,** 757–759.
3. Fujino, T., Nakagawa, N., Yuhki, K., Hara, A., Yamada, T., Takayama, K., Kuriyama, S., Hosoki, Y., Takahata, O., Takanobu, T., Fukuzawa, J., Hasebe, N., Kikuchi, K., Narumiya, S., and Ushikubi, F. (2004) Decreased susceptibility to renovascular hypertension in mice lacking the prostaglandin $I_2$ receptor IP. *J. Clin. Invest*. **114**, 805–812.
4. Mantry, P., Shah, A., and Sundaram, U. (2003) Celecoxib associated esophagitis. Review of gastrointestinal side effects from Cox-2 inhibitors [alimentary tract: case report]. *J. Clin. Gastroenterol*. **37**, 61–63.
5. Mukherjee, D., Nissen, S.E., and Topol, E.J. (2001) Risk of cardiovascular events associated with selective COX-2 inhibitors. *JAMA* **286(8),** 954–959.
6. Davies, N.M. and Jamali, F. (2004) COX-2 selective inhibitors cardiac toxicity: getting to the heart of the matter**.** *J. Pharm. Pharmaceut. Sci*. **7(3),** 332–336.
7. Cipollone, F., Rocca, B., and Patrono, C. (2004) Cyclooxygenase-2 expression and inhibition in atherothrombosis. *Arterioscler. Thromb. Vasc. Biol.* **24**, 246.
8. FitzGerald, G.A., Cheng, Y., and Austin, S. (2001). Cox-2 inhibitors and the cardiovascular system. *Expl. Rheumatol.* **19 (SUPPL. 25)**, S31-S36.

9. Wright, K.L., Weaver, S.A., Patel, K., Coopman, K., Feeney, M., Kolios, G., Robertson, D.A., and Ward, S.G. (2004) Differential regulation of prostaglandin E biosynthesis by interferon-γ in colonic epithelial cells. *Br. J. Pharmacol*. **141,** 1091–1097.
10. FitzGerald, G.A., Cheng, Y., and Austin, S. (2001) COX-2 inhibitors and the cardiovascular system. *Clin. Exp. Rheumatol*. **19**, S31–S36.
11. Reddy, B.S. and Rao, C.V. (2002) Novel approaches for colon cancer prevention by cyclooxygenase-2 inhibitors. *J. Environ. Pathol. Toxicol. Oncol*. **21**, 155–164.
12. Breyer, M., Zhang, Y., Guan, Y.F., Hao, C.M., Hebert, R.L., and Breyer, R.M. (1998) Regulation of renal function by prostaglandin E receptors. *Kidney Int*. **67**, S88–S94.
13. Raymond, C.H. (2000) Cyclooxygenase-2 in the kidney. *J. Am. Soc. Nephrol*. **11**, 2387–2394.
14. Solomon, S.D., McMurray, J.J., Pfeffer, M.A., Wittes, J., Fowler, R., Finn, P., Anderson, W.F., Zauber, A., Hawk, E., Bertagnolli, M., and Adenoma Prevention with Celecoxib (APC) Study Investigators (2005) Cardiovascular risk associated with celecoxib in a clinical trial for colorectal adenoma prevention. *N. Engl. J. Med*. **352,** 1071–1080.
15. King, S.A. (2000) The Use of NSAIDs for Geriatric Pain. *Geriatric Times* **1(2).** http://www.geriatrictimes.com/g000805.html
16. Gottlieb, S. (2001) COX 2 inhibitors may increase risk of heart attack. *Br. Med. J.* **323,** 471.
17. Mamdani, M. (2004) Cyclo-oxygenase-2 inhibitors versus non-selective non-steroidal anti-inflammatory drugs and congestive heart failure outcomes in elderly patients: a population-based cohort study. *Lancet* **363**, 1751–1756.
18. Finckh, A. and Aronson, M.D. (2005) Cardiovascular risks of cyclooxygenase-2 inhibitors: where we stand now. *Ann. Intern. Med*. **142**, 212–214.
19. Diwan, J.J. (2004) Synthesis of Eicosanoids. http://www.rpi.edu/dept/bcbp/molbiochem/MBWeb/mb2/part1/prostag.htm#cycl
20. FitzGerald, G.A. and Patrono, C. (2001) The coxibs, selective inhibitors of cyclooxygenase-2. *N. Engl. J. Med*. **345**, 433–442.
21. Mukherjee, D. and Topol, E.J. (2003) Cox-2: where are we in 2003? Cardiovascular risk and Cox-2 inhibitors. *Arthritis Res. Ther*. **5**, 8–11.
22. Drazen, J.M. (2005) COX-2 inhibitors — a lesson in unexpected problems. *N. Engl. J. Med.* **352,** 1131–1132.
23. Perazella, M.A. (2004) COX-2 Inhibitors and the Kidney. http://www.hosppract.com/issues/2001/03/ceper.htm
24. Finckh, A. and Aronson, M.D. (2005) Cardiovascular risks of cyclooxygenage-2 inhibitors: Where we stand now. *Ann. Intern. Med.* **142,** 1-4.
25. Cheng, H.-F. and Harris, R.C. (2004) Cyclooxygenases, the kidney, and hypertension. *Hypertension* **43(3),** 525–530.
26. Barraclough, D.R. et al. (2002) Considerations for the safe prescribing and use of COX-2-specific inhibitors. *Med.J. Aust*. **176(7),** 328–331.
27. Morales, E. and Mucksavage, J.J. (2002) Cyclooxygenase-2 inhibitor-associated acute renal failure: case report with rofecoxib and review of the literature. *Pharmacotherapy* **22(10),** 1317–1321.
28. Breyer, M.D. and Harris, R.C. (2001) Cyclooxygenase 2 and the kidney. *Curr. Opin. Nephrol. Hypertens*. **10**, 89–98.
29. Savage, R. (2005) Cyclo-oxygenase-2 inhibitors: when should they be used in elderly? *Drugs Aging* **22**, 185–200.
30. Ahmed, A.A., ElSeedi, H.R., Mahmoud, A.A., ElDouski, A.E.A., Zeid, I.F., and Bohlin, L. (1998) Eudesmane derivatives from Laggera crispata and Pluchea carolonesis. *Phytomedicine* **49(8),** 2421–2424.
31. Steinmeyer, J. (2000) Pharmacological basis for the therapy of pain and inflammation with nonsteroidal anti-inflammatory drugs. *Arthritis Res*. **2**, 379–385.
32. Loftin, C.D., Trivedi, D.B., and Langenbach, R. (2002) Cyclooxygenase-1–selective inhibition prolongs gestation in mice without adverse effects on the ductus arteriosus. *J. Clin. Invest.* **110,** 549–557.
33. Landers, S.J. (2005) More heart risks found for COX-2 inhibitors. http://www.ama-assn.org/amednews/index.htm
34. Gambaro, G. (2002) Strategies to safely interfere with prostanoid activity while avoiding adverse renal effects: could COX-2 and COX-LOX dual inhibition be the answer? *Nephrol. Dial. Transplant.* **17**, 1159–1162.
35. Penn Researchers Add More Evidence To Demonstrate Role Of COX Inhibitors In Heart-disease Risk. 27 /1/ 2005. http://www.uphs.upenn.edu/news/News_Releases/jan05/COX.htm .
36. Venables, J. (2004) NSAID Use and Endurance Running. Foundations of a Patient Advisory. http://www.findarticles.com/p/articles/mi_m0NHG/is_3_17/ai_n13467250

### BIOSKETCH

**Jagdish N. Sharma** is Professor and Ex-Chairman, Department of Pharmacology and Applied Therapeutics, Kuwait University, Safat, Kuwait. Dr. Sharma received his B.V.Sc. and A.H. (D.V.M.) in 1970 from Jawaharlal Nehru Agricultural University, Jabalpur, India; his M.Sc. in Pharmacology in 1973 from the All-India Institute of Medical Sciences, New Delhi, India; and his Ph.D. in Pharmacology in 1976 from the University of Strathclyde, Glasgow, Scotland. In 1995, he received an F.C.P. from the American College of Clinical Pharmacology, New York and an F.I.Biol. in 1997 from the Royal Institute of Biology, London. In addition to his many educational duties, Dr. Sharma serves as either an Editorial or Advisory Board Member, or both, for several national and international journals and conferences, and has published extensively in national and international journals and conferences. He is also a Member of the British Pharmacological Society of Great Britain. Dr. Sharma made highly significant research contributions on research studies based on pharmacological and pathophysiological roles of the kallikrein-kinin system in hypertension, cardiac hypertrophy, cardiovascular system, diabetes, respiratory system and inflammatory diseases.
